# Development of Robust and Standardized Cantilever Sensors Based on Biotin/Neutravidin Coupling for Antibody Detection

**DOI:** 10.3390/s130405273

**Published:** 2013-04-19

**Authors:** Jiayun Zhang, Hans Peter Lang, Felice Battiston, Natalija Backmann, Francois Huber, Christoph Gerber

**Affiliations:** 1 Swiss Nano Institute, University of Basel, Klingelbergstrasse 82, 4056 Basel, Switzerland; E-Mails: hans-peter.lang@unibas.ch (H.P.L.); natalija.backmann@unibas.ch (N.B.);francois.huber@unibas.ch (F.H.); christoph.gerber@unibas.ch (C.G.); 2 Concentris GmbH, Davidsbodenstrasse 63, 4056 Basel, Switzerland; E-Mail: battiston@concentris.ch

**Keywords:** cantilever, biosensor, protein, multilayer, NeutrAvidin, biotin

## Abstract

A cantilever-based protein biosensor has been developed providing a customizable multilayer platform for the detection of antibodies. It consists of a biotin-terminated PEG layer pre-functionalized on the gold-coated cantilever surface, onto which NeutrAvidin is adsorbed through biotin/NeutrAvidin specific binding. NeutrAvidin is used as a bridge layer between the biotin-coated surface and the biotinylated biomolecules, such as biotinylated bovine serum albumin (biotinylated BSA), forming a multilayer sensor for direct antibody capture. The cantilever biosensor has been successfully applied to the detection of mouse anti-BSA (m-IgG) and sheep anti-BSA(s-IgG) antibodies. As expected, the average differential surface stress signals of about 5.7 ± 0.8 × 10^−3^ N/m are very similar for BSA/m-IgG and BSA/s-IgG binding, *i.e.*, they are independent of the origin of the antibody. A statistic evaluation of 112 response curves confirms that the multilayer protein cantilever biosensor shows high reproducibility. As a control test, a biotinylated maltose binding protein was used for detecting specificity of IgG, the result shows a signal of bBSA layer in response to antibody is 5.8 × 10^−3^ N/m compared to bMBP. The pre-functionalized biotin/PEG cantilever surface is found to show a long shelf-life of at least 40 days and retains its responsivity of above 70% of the signal when stored in PBS buffer at 4 °C. The protein cantilever biosensor represents a rapid, label-free, sensitive and reliable detection technique for a real-time protein assay.

## 1. Introduction

Microfabricated silicon cantilever sensor arrays represent a powerful platform for a broad range of detection applications in physics [1], chemistry [2], material science [3], biology [4] and medicine [5]. This label-free, real-time technology makes it possible to monitor the interactions of a wide range of molecules, including proteins [6], nucleic acids [7], and small molecules [8,9]. The sensor response is mechanical bending due to absorption of molecules on the cantilever surface.

Robust field applications requested by commercial users of the cantilever technique require development of working protocols, *i.e.*, standard operation procedures (SOPs), and sensor kits to perform experiments in a reliable and reproducible way. The existence of such protocols for other sensor techniques like the quartz crystal microbalance [10], surface plasmon resonance [11] or DNA-microarrays [12] bridges the gap between scientific results achieved in the laboratory and the every-day requirements of industry. For cantilever sensors, the key for routine operation definitely lies in the availability of reliable, well-characterized cantilever arrays in terms of stable chemical functionalization and substantial shelf life to fulfill the requirements in industrial applications.

The presence of certain antibodies in individuals indicates the occurrence of diseases, in particular of various virus infections, hence, the development of antibody-detection tests is important for diagnosis and effective disease treatment, such as for human immune deficiency virus (HIV) and auto- immune diseases [13]. Using the biotin-NeutrAvidin chemistry, we developed a multilayer model biosensor for detection of bovine serum albumin (BSA)-specific antibodies. In real world application the specificity of the sensor can easily be redirected toward different kinds of antibodies.

The biotin-NeutrAvidin interaction [14] is one of the most widely used coupling mechanisms in bioconjugation chemistry. NeutrAvidin is a tetrameric protein with each subunit being capable of binding one molecule of biotin. Due to the strong binding affinity and high specificity of the interaction, NeutrAvidin is used as a transduction layer in a multilayer system to mediae between the biotin-coated surface and the biotinylated receptors, such as biotinylated BSA. Based on the biotin-NeutrAvidin interaction, we have developed a multilayer biosensor utilizing a layer of biotin-terminated poly-ethyleneglycol (PEG) undecanethiol molecules that is known to reduce non-specific binding of proteins [15]. We have studied the shelf life stability of biotin/PEG-functionalized cantilever arrays by adsorption of NeutrAvidin. The results indicate that the surface can be kept in PBS buffer for over 40 days without a major loss in sensitivity compared to freshly prepared sensors. Further, we have coupled biotinylated BSA to the NeutrAvidin/biotin/PEG-layer and measured the binding of monoclonal mouse IgG antibodies (m-IgG) and polyclonal sheep IgG antibodies (s-IgG). The average binding signals of 6.0 ± 1.0 × 10^−3^ N/m and 5.3 ± 0.4 × 10^−3^ N/m for BSA/m-IgG and BSA/s-IgG respectively showed no significant difference. The findings underline the high reproducibility of the multilayer format cantilever sensor for antibody detection. The results obtained allow provision of well-tested protocols and measurement kits for reliable and reproducible cantilever sensing experiments on a variety of consumer-modifiable chemical platforms.

## 2. Experimental Section

All experiments were performed using commercially available static mode cantilever arrays with eight identical silicon cantilevers having the following dimensions: 500 μm in length, 100 μm in width, and 0.9 μm in thickness (IBM Research GmbH, Rüschlikon, Switzerland, and Concentris GmbH, Basel, Switzerland).

### 2.1. Reagents

NeutrAvidin was obtained from Pierce (Rockford, IL, USA). Bovine serum albumin (BSA) and biotinylated bovine serum albumin (biotinylated BSA) were purchased from Sigma-Aldrich (St. Louis, MO, USA). A recombinant biotinylated maltose binding protein (bMBP) was kindly provided by A. Plückthun (Department of Biochemistry, University of Zurich, Zurich, Switzerland). 1-Mercapto-11-undecyl)-tetra-(ethylene glycol) and biotin-terminated tri-(ethylene glycol)-undecanethiol (SH-C_11_-(PEG)_3_-biotin) were acquired from Asemblon Inc. (Seattle, WA, USA). 2-[Methoxy(polyethyleneoxy)propyl] trimethoxysilane was purchased from abcr GmbH & Co. KG (Karlsruhe, Germany). Mouse monoclonal anti-bovine albumin antibody (mouse anti-BSA, m-IgG) and sheep polyclonal anti-bovine albumin antibody (sheep anti-BSA, s-IgG) were bought from Biotrend Chemikalien GmbH (Köln, Germany) and Immunology Consultants Laboratory, Inc. (Portland, OR, USA), respectively. All protein solutions were prepared in phosphate-buffered saline (0.01M PBS; 137 mM NaCl, and 2.7 mM KCl, pH 7.4).

### 2.2. Cantisens^®^ Research Cantilever Array System

All measurements were performed on the commercial Cantisens^®^ cantilever sensor [16,17] measurement platform (Concentris GmbH, Basel, Switzerland) equipped with a liquid cell of 5 μL. Liquid samples were injected using an automated liquid handling system. A functionalized array was mounted on the array holder cartridge in the liquid cell in such a way that the cantilevers face the incoming laser beam emitted from time-multiplexed VCSELs (vertical cavity surface-emitting lasers, Avalon Photonics, Zürich, Switzerland), allowing *in-situ* monitoring of cantilever deflection. A LabView program (National Instruments, Austin, TX, USA) controls liquid handling via a syringe pump and a two-position injection valve that enables sample injection into the cell by switching liquid flow between sample port through an external sample loop and buffer flow, as well as temperature regulation at 20.00 ± 0.01 °C, data acquisition and data processing. Data analysis was performed with the Concentris Dataviewer software (Concentris GmbH).

### 2.3. Cantilever Preparation

The preparation of cantilever sensor arrays has been described previously [18]. Prior to use, cantilever arrays undergo the following procedure: (1) cleaned twice in freshly prepared piranha solution (H_2_O_2_:H_2_SO_4_ = 1:1) for 30 min; (2) rinsed with Nanopure water and absolute ethanol; (3) dried on a hot plate at 75 °C. To prevent non-specific binding on the bottom side of the cantilever, the array was immersed into a 10 mM 2-[methoxy(polyethyleneoxy)propyl]trimethoxysilane solution in ethanol for 45 min, washed three times using absolute ethanol and dried with argon. Then a 2 nm thick titanium layer and a 20 nm thick gold layer were deposited onto the top side of cantilever using an electron beam evaporator (EVA300, Alliance Concept, Cran Gevrier, France). Each of the freshly prepared gold-coated cantilever arrays was functionalized with 4–6 probes for target capture and 2–4 non-specific references, respectively. The functionalized array was rinsed using 10 mM PBS buffer and stored at +4 °C.

### 2.4. Pre-Functionalization of Cantilevers with a SH-C_11_-(PEG)_3_-Biotin Layer

**Figure 1.**
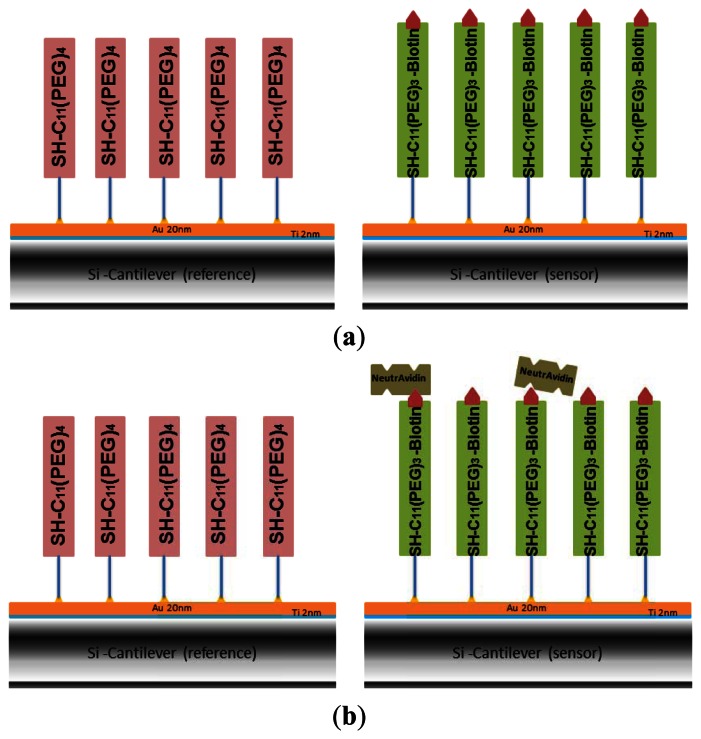

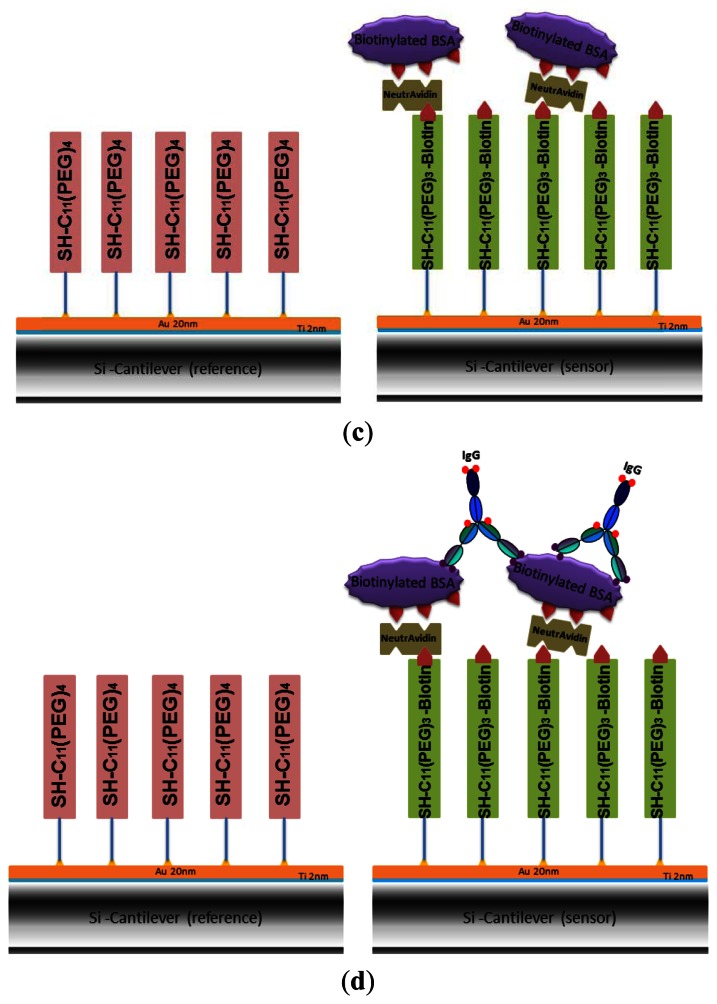
Schematics of cantilever functionalization: (**a**) gold-coated cantilevers were functionalized with a self-assembled monolayer of SH-C_11_-(PEG)_3_-biotin as a sensor to detect NeutrAvidin. Other cantilevers were coated with SH-C_11_-(PEG)_4_ inert to NeutrAvidin serving as reference. (**b**) Subsequent functionalization with NeutrAvidin. (**c**) Adsorption of biotinylated BSA forms a multilayer for capturing antibodies. (**d**) Scheme depicting how antibodies are captured by the multilayer cantilever biosensor.

The gold-coated cantilever array was first functionalized with undecanethiol, containing three ethylene glycol units and a biotin group (SH-C_11_-(PEG)_3_-biotin) by using microcapillaries filled with a 50 μM solution of SH-C_11_-(PEG)_3_-biotin in ethanol for 30 min (shown in green in Figure 1(a)). Since only differential measurements provide reliable results [19], some of the cantilevers have been functionalized as reference cantilevers by self-assembly of a SH-C_11_-(PEG)_4_ monolayer (shown in pink in Figure 1(a)), which is inert to NeutrAvidin in subsequent immobilization. The array was rinsed with absolute ethanol and Nanopure water and stored at +4 °C in PBS. In this work, several pre-functionalized arrays were also stored in argon at +4 °C for evaluation of optimized storage conditions.

### 2.5. Formation of a Multilayer System through Subsequent Functionalization of Cantilevers with NeutrAvidin and Biotinylated BSA

The biotin groups attached on the gold-coated cantilever surface were used to capture NeutrAvidin by immersing the whole array in 1.5 μM NeutrAvidin solution in PBS for 20 min (Figure 1(b)). The array was rinsed three times with buffer. The process of NeutrAvidin immobilization was also carried out in the cantilever device by injecting the same concentration of NeutrAvidin into the liquid cell at a rate of 0.83 μL/s. Data on biotin-NeutrAvidin binding was recorded. No significant difference to the results from immersing the whole array was observed, detailed results are shown in the Supplementary Information. Afterwards the cell was flushed using PBS buffer to remove unbound protein. Biotinylated BSA was coupled to the NeutrAvidin layer (Figure 1(c)) by dipping the cantilever array into a 1.5 μM solution of biotinylated BSA in PBS buffer for 20 min. To remove loosely adsorbed biotinylated-BSA, the surface was rinsed three times with PBS.

### 2.6. Cantilever Deflection Measurements

The functionalized cantilever array was mounted into the liquid measurement cell, exposed to the PBS buffer flow of 0.83 μL/s, and the system was equilibrated at a temperature of 20 °C. To test the mechanical homogeneity of each cantilever and the direction of absolute deflection bending signals, a heating test was performed by rising the temperature from 20 to 22 °C for 2 min in the measurement cell (data not shown). Due to the bimetallic effect of gold-coated microcantilevers, the observed negative deflection signal corresponds to downward bending of the cantilever (compressive stress, bending away from the gold coating). For detection of binding, 500 μL of the target solution were injected into the sample loop while buffer flows directly through to the liquid cell, and subsequently into the measurement cell by switching the two position injection valve. Differential deflection was calculated by subtraction of the responses of the reference cantilevers from the deflection signal of the sensor cantilevers.

### 2.7. Specificity of Cantilever Sensors

In order to test the specificity of the biotinylated BSA cantilevers, the biotinylated maltose binding protein bMBP was chosen as a control protein in the detection of mouse monoclonal IgG. The gold-coated cantilever array was first functionalized with undecanethiol, containing three ethylene glycol units and a biotin group [SH-C_11_-(PEG)_3_-biotin] by immersing into a 50 μM solution of SH-C_11_-(PEG)_3_-biotin in ethanol for 30 min, rinsing with absolute ethanol and Nanopure water, followed by immersing into 1.5 μM NeutrAvidin solution. The surface was then washed with buffer to remove nonspecifically adsorbed molecules. In this experiment, as the reference cantilevers, *i.e.*, four of 8 cantilevers within array were functionalized with the control protein–bMBP, and the other four sensor cantilevers were still functionalized with biotinylated BSA (Figure 1(c)). The specificity of the array was explored by monitoring the response of the biotinylated BSA on the cantilevers with injection of 50 μg/mL m-IgG.

## 3. Results and Discussion

### 3.1. Assessment of Cantilevers Pre-Functionalized with Biotin

**Figure 2.**
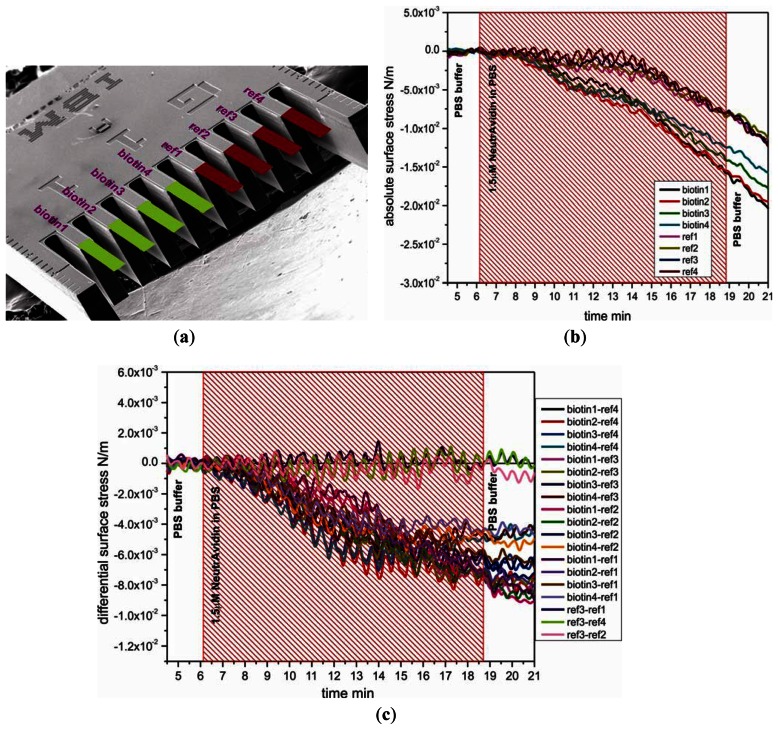
(**a**) Pre-functionalization of cantilevers coated with SH-C_11-_(PEG)_3_-biotin on cantilever 1-4 (biotin1 to biotin4, shown in green), and with SH-C_11_-(PEG)_4_ on cantilever 5-8 (ref1 to ref4, shown in red, randomly selected). (**b**) Absolute bending signal after specific interaction of biotin and NeutrAvidin (hatched area) displaying different reactivity for sensors and reference cantilevers. (**c**) Differential bending signal showing a very reproducible negative signal that corresponds to compressive stress building up on the sensor cantilever. Differences between reference cantilevers show no signal. The periodic oscillation observed in the signals is due to the injection process (imperfection of the syringe pump gear).

To study the functionality and stability of the SH-C_11_-(PEG)_3_-biotin layer on cantilevers, a 1.5 μM solution of NeutrAvidin in PBS was directly injected into the measurement cell of the cantilever device. Figure 2(b) shows the absolute responses to injection of NeutrAvidin, which are divided into two groups, the sensor group and the reference group. The absolute surface stress of sensor cantilever group for specific binding of biotin and NeutrAvidin was observed to produce more downward bending than for reference cantilever group, demonstrating that immobilization of NeutrAvidin onto SH-C_11_-(PEG)_3_-biotin layers was efficient. The differential measurement between sensor cantilevers (biotin1, 2, 3, and 4) and reference cantilevers (ref1, 2, 3, and 4) displayed in Figure 2(a) clearly shows the generation of compressive surface stress of around −6.4 ± 0.5 × 10^−3^ N/m associated with the binding of NeutrAvidin to SH-C_11_-(PEG)_3_-biotin. The magnitudes of the signals are very similar, shown e.g., for the twelve pairs of reference and sensor cantilevers displayed in Figure 2(c). As expected, the difference between three pairs of reference cantilevers produces no signal.

### 3.2. Shelf life of Cantilevers Pre-Functionalized with Biotin

**Figure 3.**
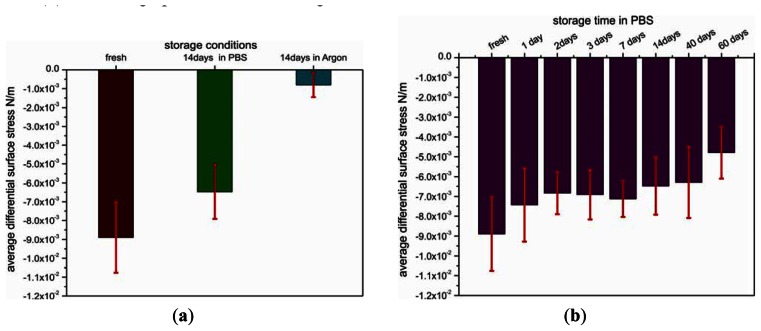
Bar graphs showing the stability of the pre-functionalized biosensor arrays. Each bar represents the average of differential surface stress with standard deviations produced by the response to 1.5μM of NeutrAvidin. (**a**) Stability in various storage environments. (**b**) shelf-life graph for different storage durations in PBS.

The important issue of shelf life has been addressed by studying the temporal and environmental stability of pre-functionalized cantilevers. For this reason, the cantilevers coated with SH-C_11_-(PEG)_3_-biotin were stored under different environmental conditions, such as argon atmosphere or PBS buffer (+4 °C), for different time periods. The functional stability of the biotin-layer was tested under the conditions mentioned before by measuring the differential binding response to injection of a 1.5 μM solution of NeutrAvidin in PBS (Figure 3(a)).

Differential surface stress signals from cantilever arrays stored for 14 days and others tested immediately after coating with a biotin layer are very distinct. The lowest signal of about −0.8 ± 0.6 × 10^−3^ N/m corresponding to compressive surface stress was observed for the cantilever array pre-functionalized with biotin that was stored in argon for 14 days, yielding only 11% of the value (−8.9 ± 1.8 × 10^−3^ N/m) obtained on the freshly coated array. In contrast, by storage in PBS buffer instead of argon for 14 days, the signal is only reduced to 73%.

As storage in PBS (+4 °C) produced the best results which is in agreement with other reports [20,21], we used the same environmental conditions to test the shelf life of the SH-C_11_-(PEG)_3_-biotin-coated cantilevers. The arrays were tested after 1, 2, 3, 7, 14, 40 and 60 days, respectively (Figure 3(b)). After one day of storage, the differential deflection signal dropped to 84% of the value obtained for freshly prepared arrays and consecutively remained above 70% over 40 days. Finally, after 60 days, the signal was only about half as large as compared to freshly prepared arrays.

The results obtained reveal a relatively long shelf life of the biotin-functionalized arrays providing a promising platform for the development of a commercial assay kit for detection of proteins. The arrays can be stored for at least 40 days in buffer without major loss of sensitivity and selectivity. It turned out that argon atmosphere alone is not sufficient to maintain the functional stability of cantilevers pre-coated with biotin due to partial deterioration of the SH-C_11_-(PEG)_3_-biotin layer preventing NeutrAvidin binding.

### 3.3. Antigen Platform for Antibody Detection

In a final step we demonstrate the usability of the multilayer sensor for the detection of antibodies. As a model system (Figure 1(d)) we selected BSA and two different BSA-specific antibodies, a monoclonal mouse (m-IgG) or polyclonal sheep (s-IgG) antibody. Biotinylated BSA was immobilized by incubating the NeutrAvidin cantilever surface in 1.5 μM biotinylated BSA solutions (schematics shown in Figure 2(c)). An antibody dilution in PBS was injected and the specific binding of antibody and antigen were monitored *in situ* during the measurement.

#### 3.3.1. Specificity of the Biotinylated BSA Sensing Cantilever to Antibody

**Figure 4.**
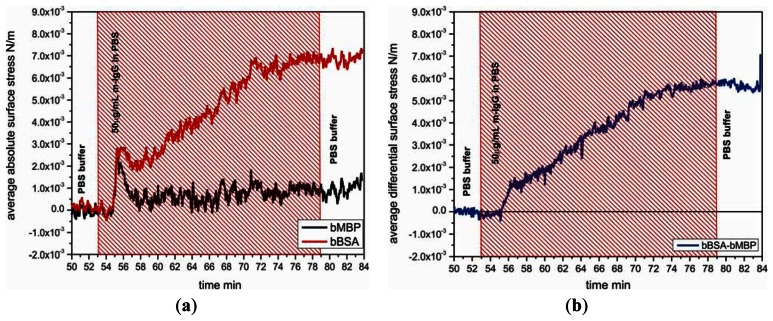
Specificity test of the biotinylated BSA layer on cantilevers to m-IgG. (**a**) The average absolute bending responses of different protein coated cantilevers upon injecting of m-IgG (red hatched area) displaying different binding affinity. (**b**) The average differential bending signal of BSA to m-IgG is evaluated by subtracting of the bending signal of bMBP to IgG.

Specificity of the biotinylated BSA layer on cantilevers was investigated by testing the binding response to m-IgG 50 μg/mL comparing with the response between the biotiylated maltose binding protein and m-IgG. Figure 4 shows the nanomechanical response of bBSA coated sensor cantilever as function of time with injection of m-IgG. The binding reaction between bBSA and m-IgG generates a tensile surface stress which leads the sensor cantilever to bend upward with respect to a reference cantilever, giving rise to a differential surface stress up to 5.8 × 10^−3^ N/m. It reveals that the affinity of bBSA to m-IgG is significantly higher than that of bMBP to m-IgG.

#### 3.3.2. Sensitivity of the Biotinylated BSA sensing Cantilever to Antibody

**Figure 5.**
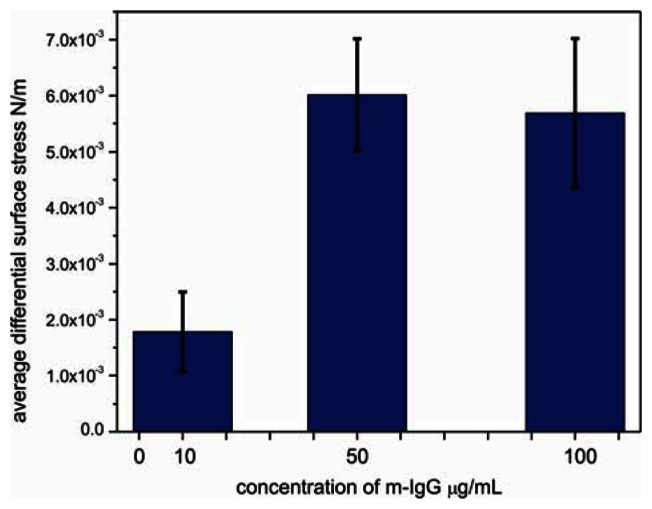
The average differential surface stress of sensor cantilever exposed to three various concentrations of m-IgG in separate experiments.

To determine the sensitivity of sensor cantilever to antibody, the responses of bBSA cantilevers were tested by performing the experiments at the lowest reported detectable concentration [22] of 10 μg/mL, at an intermediate concentration of 50 μg/mL and at a high concentration of 100 μg/mL of m-IgG (Figure 5). The surface stress of bBSA to m-IgG obtained at low concentration is considerably smaller than at the intermediate concentration of 50 μg/mL. When the cantilever was exposed to 10μg/mL, the differential response was observed to be 1.7 × 10^−3^ N/m; at 50 μg/mL, the binding between bBSA and m-IgG leads to a considerable increase in the signal to a value of 5.7 × 10^−3^ N/m. However, upon injecting 100 μg/mL of m-IgG, the bending signal is unchanged (5.5 × 10^−3^ N/m), suggesting that the surface of the cantilever was saturated above concentrations of 50 μg/mL. The small change in bending signal might be associated with a conformational rearrangement of bound antibody.

#### 3.3.3. Reproducibility of the Biotinylated BSA Sensing Cantilever to an Antibody

**Figure 6.**
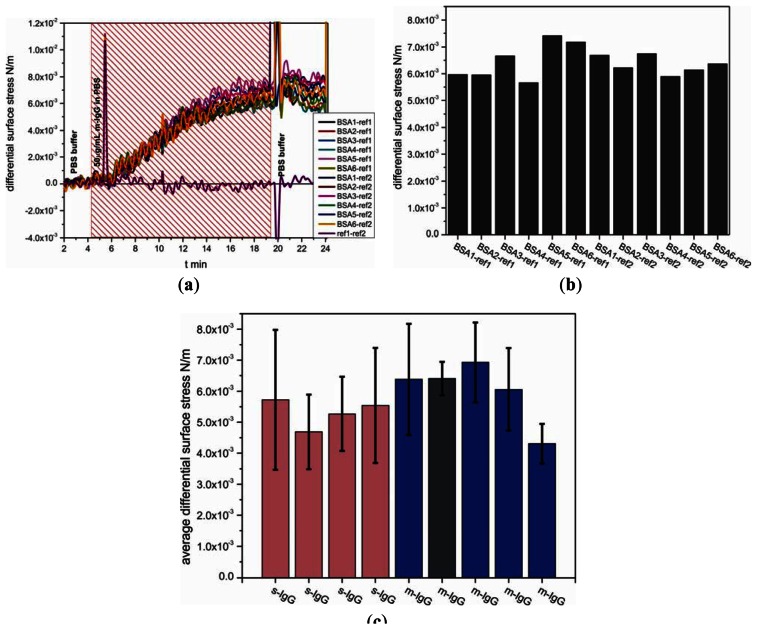
(**a**) shows individual measurement curves analyzed for one out of nine cantilever arrays. In this experiment, the sensor cantilevers were immobilized with bBSA as the sensing layer, only two cantilevers served as reference cantilevers. The difference in responses between a sensor and a reference cantilever shows that the sensor cantilevers bent upward (producing a positive signal), corresponding to tensile surface stress. (**b**) Bar graph showing the individual differential bending signal values for each curve. (**c**) Each bar represents the average independent measurements of the difference in responses of a sensor and a reference cantilever. Measurements were carried out on nine different arrays (displayed by nine bars). Recognition between bBSA and antibody (either s-IgG (pink bars) or m-IgG (blue including dark grey bars)) produced mean deflections that do not differ significantly for both the 4 s-IgG arrays and the 5 m-IgG arrays. The dark grey bar displays the average of differential signal corresponding to the data shown in (b).

The measurements were carried out on nine separate cantilever arrays comprising total 38 individual sensors and 27 references cantilevers, *i.e.*, 65 out of 72 cantilevers, 112 differential measurement curves in total. In a separate experiment for detecting antibodies, functionalized biotinylated BSA cantilevers were exposed to m-IgG at a concentration of 50 μg/mL. Figure 6(a,b) show measurement curves for one out of nine analyzed arrays. To evaluate the binding performance of the antigen cantilever arrays, the average differential surface stress (Figure 6(a)) and standard deviation values (Figure 6(c), black error bars) were calculated from all possible combinations between sensor and reference cantilevers within each individual array shown in Figure 6(b). The data compiled in Figure 6(c) represent averaged differential bending signals obtained for each of nine individual cantilever arrays. For testing the reproducibility with different sensor cantilevers from m-IgG and s-IgG, we observed no significant difference between the binding of m-IgG (shown in blue bars including dark grey bar) and s-IgG (shown in pink bars), as expected for the conditions chosen. The results clearly demonstrate that the binding between IgG and BSA is reproducible tensile surface stress with an average differential surface stress of about 5.7 ± 0.8 × 10^−3^ N/m.

In summary, antibody detection using the multilayer sensor has proven reliability, reproducibility and robustness of the technique.

## 4. Conclusions

We have developed a protein cantilever biosensor based on a multilayer platform consisting of a pre-functionalized cantilever surface with a biotin-terminated PEG monolayer and a NeutrAvidin layer for subsequent binding of a biotinylated antigen, which serves to capture an antibody. The average observed signal has amounted to 5.7 ± 0.8 × 10^−3^ N/m. The important issue of shelf life is addressed by studying the temporal and environmental stability of pre-functionalized cantilevers. After storage in PBS buffer at 4 °C for 40 days, pre-functionalized cantilevers have kept their functionality by still showing a response above 70%. The reliability, reproducibility and robustness of the multilayer sensor have been verified. This finding is crucial for the development of a commercial assay kit. Sufficient measurement statistics is indeed a prerequisite for a robust sensing device.

## Supplementary Material
