# Supplementary material for: Development of Robust and Standardized Cantilever Sensors Based on Biotin/Neutravidin Coupling for Antibody Detection
Source: Sensors (Basel). 2013 Apr 19;13(4):5273–85. doi: 10.3390/s130405273 (PMC3673136; doi:10.3390/s130405273)
Supplement: Supplementary file 1 [file sensors-13-05273-s001.pdf]

OPEN ACCESS

sensors

ISSN 1424-8220

www.mdpi.com/journal/sensors

Supplementary Information

Development of Robust and Standardized Cantilever Sensors Based on Biotin/Neutravidin Coupling for Antibody Detection. *Sensors* 2013, *13*, 5273-5285

Jiayun Zhang [1,*], Hans Peter Lang [1], Felice Battiston [2], Natalija Backmann [1], Francois Huber [1] and Christoph Gerber [1]

[1] Swiss Nano Institute, University of Basel, Klingelbergstrasse 82, 4056 Basel, Switzerland; E-Mails: hans-peter.lang@unibas.ch (H.P.L.); natalija.backmann@unibas.ch (N.B.); francois.huber@unibas.ch (F.H.); christoph.gerber@unibas.ch (C.G.)

[2] Concentris GmbH, Basel, Davidsbodenstrasse 63, 4056 Basel, Switzerland; E-Mail: battiston@concentris.ch

* Author to whom correspondence should be addressed; E-Mail: jiayun.zhang@unibas.ch; Tel.: +41-61-267-3769; Fax: +41-61-267-3784.

Figure A. Comparison of responses of IgG with multilayer which was formed using different functionalization methods. Orange bars: subsequent functionalization in the cantilever device by injecting 1.5 μM NeutrAvidin solution into the measurement cell; violet bars: subsequent functionalization in 100 μL of container by immersing the whole array in the same concentration of NeutrAvidin solution.

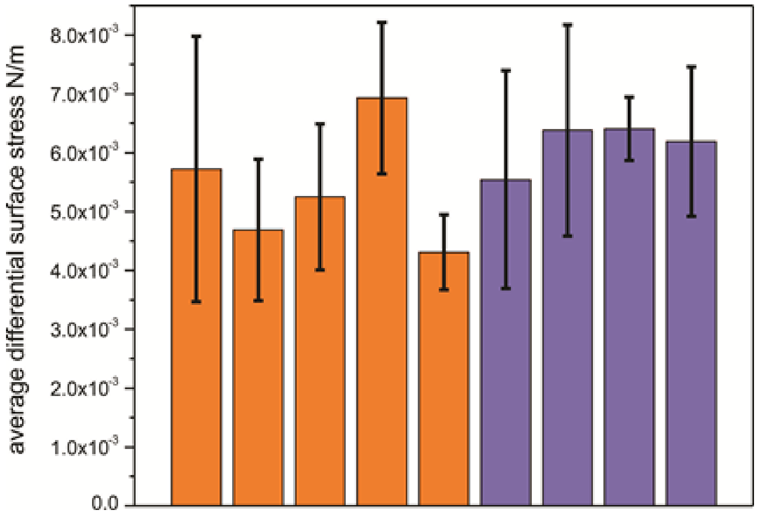

© 2013 by the authors; licensee MDPI, Basel, Switzerland. This article is an open access article distributed under the terms and conditions of the Creative Commons Attribution license (http://creativecommons.org/licenses/by/3.0/).
